# Alterations to Kidney Physiology during Cardiopulmonary Bypass—A Narrative Review of the Literature and Practical Remarks

**DOI:** 10.3390/jcm12216894

**Published:** 2023-11-01

**Authors:** Jakub Udzik, Jerzy Pacholewicz, Andrzej Biskupski, Paweł Walerowicz, Kornelia Januszkiewicz, Ewa Kwiatkowska

**Affiliations:** 1 Department of Cardiac Surgery, Pomeranian Medical University, Powstancow Wielkopolskich 72, 70-111 Szczecin, Poland; jerzy.pacholewicz@pum.edu.pl (J.P.); a.biskupski@vp.pl (A.B.); pawel.walerowicz@pum.edu.pl (P.W.); 2 Department of Anesthesiology, Intensive Care and Acute Intoxications, Pomeranian Medical University, Powstancow Wielkopolskich 72, 70-111 Szczecin, Poland; k.januszkiewicz@spsk2-szczecin.pl; 3 Clinical Department of Nephrology, Transplantology and Internal Medicine, Pomeranian Medical University, 70-111 Szczecin, Poland; ewakwiat@gmail.com

**Keywords:** cardiac surgery, clinical physiology, cardiopulmonary bypass, acute kidney injury

## Abstract

Introduction: According to different authors, cardiac surgery-associated acute kidney injury (CSA-AKI) incidence can be as high as 20–50%. This complication increases postoperative morbidity and mortality and impairs long-term kidney function in some patients. This review aims to summarize current knowledge regarding alterations to renal physiology during cardiopulmonary bypass (CPB) and to discuss possible nephroprotective strategies for cardiac surgeries. Relevant sections: Systemic and renal circulation, Vasoactive drugs, Fluid balance and Osmotic regulation and Inflammatory response. Conclusions: Considering the available scientific evidence, it is concluded that adequate kidney perfusion and fluid balance are the most critical factors determining postoperative kidney function. By adequate perfusion, one should understand perfusion with proper oxygen delivery and sufficient perfusion pressure. Maintaining the fluid balance is imperative for a normal kidney filtration process, which is essential for preserving the intra- and postoperative kidney function. Future directions: The review of the available literature regarding kidney function during cardiac surgery revealed a need for a more holistic approach to this subject.

## 1. Introduction

Recent data estimate that about a million patients undergo cardiac surgery every year [1]. Of these, 20 to even 50%, according to some authors, will suffer from cardiac surgery associated acute kidney injury (CSA-AKI) [2,3,4]. Apart from its high incidence, this complication bears severe consequences for the patient’s health. It increases postoperative morbidity and mortality [2,5,6], and can result in the development of chronic kidney disease (CKD) [4,7].

Cardiopulmonary bypass (CPB) is a technique that allows one to conduct most valvular procedures and coronary surgeries while having the heart in diastole. It preserves systemic blood circulation and provides very effective blood oxygenation. However, it is also the reason for many complications associated with heart surgery, such as excessive blood loss, systemic inflammatory response syndrome (SIRS), cerebral stroke, and AKI [8]. If not for the use of the CPB, cardiac surgery might have a complication rate similar to prolonged non-cardiac surgeries. Moreover, the off-pump cardiac surgeries have the advantage of shorter operation time (no time spent on cannulation, decannulation, and reperfusion) and no need for great vessels’ cannulation, which reduces the surgical trauma. Nonetheless, CPB enables the surgeon to perform operations involving the opening of the aorta and/or chambers of the heart (e.g., valvular surgery, correction of the septal defects within the heart). The most common cardiac surgery—coronary artery bypass grafting (CABG) [9]—can be performed without the use of CPB, but it renders the operation technically difficult and is not preferred by most surgeons [10]. All things considered, CPB has its disadvantages and related complications but is otherwise indispensable to cardiac surgery.

In order to understand how the kidneys are being damaged during cardiac surgery, one has to realize all the alterations to the functioning of the human body once extracorporeal circulation is initiated. Cardiac output (CO) is replaced with the CPB pump flow; therefore, the perfusionist controls the blood flow through the entire body, as well as the pressure within the arterial system. Priming solution filling the CPB circuit fuses with the patient’s blood, causing hemodilution and decreasing the oxygen-carrying capacity of the blood (CaO_2_). Both priming and cardioplegic solutions used to stop and protect the heart are highly osmotic fluids which increase the osmolality of the intravascular and interstitial fluid. Leukocytes in the blood come in contact with the artificial surface of the CPB tubing system, which causes their activation and leads to the induction of the inflammatory response [11]. Should any organ or tissue become ischemic during the CPB, it will suffer from ischemia-reperfusion injury (IRI) after weaning from it.

Three major factors can cause AKI: prerenal, renal, and postrenal. The prerenal causes dominate during cardiac surgery, followed by the renal factors. In this review, the authors discuss the alterations to renal physiology during the CPB and address the key factors influencing kidney function during and after cardiac surgery. This review aims to highlight the importance of perioperative care in the prevention of CSA-AKI and to provide practical suggestions on nephroprotective strategies during cardiac surgery.

## 2. Relevant Sections

### 2.1. Systemic and Renal Circulation

Before the beginning of CPB, the tubing system needs to be connected to the patient’s circulatory system. The venous cannula (or cannulas) is placed in the vena cava and usually passes through the right atrium. This compromises the blood’s return to the heart and decreases CO before CPB begins. Together, with a decrease in CO, there is a decrease in the mean arterial pressure (MAP). Though the time when the heart and the great vessels are cannulated but the CPB is not yet started is usually short, it is essential to maintain an adequate CO and MAP at all times. Even a period of hypotension (defined as MAP < 65 mmHg) as short as 10 min increases the risk of kidney injury [12]. Possible interventions at this stage of surgery involve raising the patient’s legs (to increase preload) or starting a low-dose adrenaline infusion (≤0.05 µg/kg/min).

There are three components of effective circulation: blood flow, CaO_2_, and perfusion pressure. Without the first two, the oxygen delivery (DO_2_) to the tissues will be compromised. Most standard CPB protocols include the pump flow rate of 2.4–2.8 L/min/m^2^ for normothermic CPB [13]. There is, however, an increasing number of scientific evidence that perfusion during the CPB should be goal-directed (goal directed perfusion—GDP) [14,15,16]. The CaO_2_ decrease associated with hemodilution varies between the patients, so the pump flow should be adjusted for each patient to maintain an adequate DO_2_. This is the essence of the GDP approach—adjusting blood flow (CPB pump performance) to reduced CaO_2_ to maintain desired DO_2_. Some researchers advocate that the DO_2_ indexed for the body surface area (iDO_2_) should be kept above a critical threshold in order to prevent AKI. De Somer et al. [16] determined it to be 262 mL/min/m^2^, and Mukaida et al. [15] set it at 300 mL/min/m^2^. Ranucci et al. [17] also proved that maintaining an iDO_2_ ≥ 280 mL/min/m^2^ during the CPB reduces CSA-AKI incidence. Srey et al. [18] applied the idea of Ranucci and his team but simplified the method. Continuous iDO_2_ monitors used by Ranucci are an expensive tool. For this reason, Srey and his team devised a formula that allows for easy calculation of the safe minimal flow rate, knowing the patient’s body surface area (BSA) and current hemoglobin level. The method assumes the nadir iDO_2_ of 280 mL/min/m^2^ and is an easy and affordable solution that can improve the postoperative outcome regarding kidney function.

Perfusion pressure is a derivative of blood flow and systemic vascular resistance (SVR) [19]. The latter usually decreases during the CPB [20] due to loss of the pulsatile blood flow [21], and also some biochemical and immunological alterations known as the vasoplegic syndrome [22,23]. The pulse wave is initiated by the left ventricular stroke volume stretching the elastic wall of the aorta [24]. This wave of recurring expansion and contraction of the arterial wall travels down to the smallest arterioles thanks to the elastic fibers of the tunica media. The force exerted on the intravascular fluid by these fibers is an essential component of the SVR. Thus, when a continuous flow of the CPB pump is applied, the SVR decreases. A full description of the pathogenesis of vasoplegic syndrome exceeds the scope of this article. It will suffice to say that it involves, among other factors, leukocyte activation, cytokine release (such as IL-6 or TNF-α), and an increase in nitric oxide production in the epithelium [22]. All these factors promote vasodilation, which in such cases can be resistant to vasopressor agents. Another factor that exacerbates vasoplegia during cardiac surgery is the influence of general anesthesia [25,26]. This factor is not unique to cardiac surgery but is especially pronounced here as high doses of fentanyl are used during this type of procedure, which may enhance vasodilation [27,28,29].

If the CPB pump flow is not high enough to compensate for decreased SVR, the pressure within the arterial tree will also decrease. In some medical professionals, there is a notion that systemic pressure during the CPB is of secondary importance as the CPB pump provides an adequate blood flow, and there is a very effective gas exchange in the oxygenator. This notion is not true. Certain areas of the circulatory system are highly dependent on blood flow pressure. Such areas involve cerebral [30] and renal arteries [31]. The anatomy of the renal arterial system differs from the other organs in the human body. The final branches of the renal artery—the afferent arterioles—divide and form the capillary net of the kidney glomerulus [32]. Subsequently, they reassemble into the efferent arteriole instead of forming the postcapillary veins. The efferent arterioles then follow the ascending limb of the Henle’s loop and divide again into capillaries surrounding the tubules. The capillaries then reassemble into veins, which arise alongside the descending limb of the Henle’s loop and confluence into greater vessels. Only a small percentage of arterial blood bypasses the glomerulus and is shunted directly into the postglomerular arteries [33]. This unique structure of the kidney vessels (capillaries reassembling into arteries) enables the creation of an outwardly directed pressure gradient within the kidney glomerulus—the effective filtration pressure (EFP). The efferent arterioles’ diameter, which is smaller than the afferent arterioles, facilitates this process. This anatomical dependency between the glomerular and peritubular vessels results in the blood flow through the peritubular vessels being almost entirely dependent on the outflow from the efferent arterioles.

The afferent and efferent arterioles are the guardians of the EFP. The afferent arteriole is equipped with a pressure sensor known as the juxtaglomerular cells [34]. Should the blood pressure within the afferent arteriole decrease, these cells will release renin, the first enzyme of the renin-angiotensin-aldosterone (RAA) axis. The RAA system is activated by low systemic pressure and is set on raising it to normal values. The threshold pressure value that activates the juxtaglomerular cells is 70–90 mmHg, according to different authors [31,35,36]. Brzozowski et al. [36] state that renin-release increases when the blood pressure falls below 90 mmHg, and that serum renin concentration doubles with every 5 mmHg of the pressure drop. Renin is an enzyme that converts Angiotensinogen to Angiotensin I (Ang I), which is later converted to Angiotensin II (Ang II) [37]. Ang II is a powerful systemic vasopressor, but it also has a particular effect on the kidneys. It constricts both the afferent and efferent arterioles. However, there is a more significant increase in the efferent arteriole resistance [38] due to its smaller resting diameter [39] and greater angiotensin receptors’ density in the efferent arteriole [40]. The net effect of this action is a rise in the EFP and a decrease in blood flow to the postglomerular vessels. The experimental mammalian studies show that a steady state response of the efferent arteriole to the Ang II is a 30% diameter reduction [41]. This means an over 4-fold increase in vascular resistance. Combined with reduced in-flow (due to afferent arteriole constriction), the blood flow to the postglomerular vessels becomes markedly decreased.

In their study, Küllmar et al. [42] demonstrated that increased plasma renin concentration was associated with cardiovascular instability and a higher incidence of CSA-AKI after cardiac surgeries. Lannemyr et al. [43] conducted a study involving renal vessel catheterization and concluded that renal perfusion decreases during the CPB due to renal vasoconstriction. The reason for this result is most probably the MAP value of 60–80 mmHg that was maintained in this study, which was probably below the kidneys’ autoregulatory threshold and provoked renin release.

The tubules are a very metabolically active region of the kidney, as numerous ATP-dependent ion transporters are located within their walls [44]. The cortical tubules can meet this high energy demand thanks to the high blood flow in this area of the kidney [45]. The medullary tubules, however, have a considerably lower blood supply—they receive 10–15% of the total renal blood flow (RBF). The efferent arterioles originating from juxtamedullary nephrons (15% of the total nephrons’ count [46]) penetrate the medulla as descending vasa recta (DVR). They divide into the capillaries and reassemble into the ascending vasa recta (AVR), which confluence with the cortical veins. The blood flow through the medulla is restricted [47] in order to allow for an effective substance exchange between the intratubular fluid and the intravascular fluid and also to prevent the washing out of the medullary osmotic gradient [45]. The parallel arrangement of the DVR and AVR necessary for countercurrent substance exchange is also the reason for oxygen shunting from the DVR to AVR, as some of the oxygen that diffuses from the DVR is not absorbed by the tubular cells but returns to the AVR [33].

There is one other important reason for maintaining sufficient perfusion pressure during the CPB. Most patients undergoing cardiac surgery are elderly, and coronary artery disease is still the most common reason for surgical intervention [9]. Suppose the patient has highly advanced atherosclerosis within the coronary arteries. In that case, it is justified to assume that he or she probably has atherosclerotic lesions in other regions of the arterial tree [48]. Renal artery stenosis more significant than 50–60% can significantly impair RBF [49], which would only worsen during the CPB. Atherosclerotic lesions cause a local increase in vascular resistance; sufficient flow pressure is required to overcome this resistance. Because of the loss of pulsation in the vascular bed during the CPB, the peak pressures achieved within the arterial system are lower than normal at the same MAP values. This is yet another argument in favor of keeping high perfusion pressure during the CPB, especially if the patient has a history of disseminated atherosclerosis. A patient’s MAP before the induction of general anesthesia can be a helpful indicator of what MAP value the patient requires.

To summarize this section, during the CPB, the kidneys need both the adequate iDO_2_ and blood pressure for proper functioning. According to recent studies, the iDO_2_ of 260–300 mL/min/m^2^ seems to be the lower threshold value necessary to maintain normal kidney oxygenation. The lower MAP threshold appears to be 70–90 mmHg. Below this MAP value, the kidneys start to release renin to raise the systemic blood pressure and maintain the EFP. This significantly reduces blood flow to the peritubular vessels and increases the risk of renal ischemia. This risk is especially high within the kidney medulla, as it is a highly metabolically active region and blood flow in this area is already limited under normal conditions. Adjusting the CPB pump flow is the simplest and most efficient method to obtain an adequate iDO_2_ and perfusion pressure. It brings an instant effect, can be easily modified if needed, and mimics a physiological response of the organism to decreased CaO_2_.

### 2.2. Vasoactive Drugs

#### 2.2.1. Norepinephrine

Norepinephrine is an agonist of alpha-1 and beta-1 adrenergic receptors [50]. Apart from small doses (<0.03 µg/kg/min), norepinephrine’s alpha agonism predominates and it acts as a potent vasopressor, not an inotrope. Norepinephrine increases the SVR and afterload, but it also influences the venous portion of the vascular system. Constricting the small veins decreases the volume of blood stored within the venous system and increases the preload. This action of norepinephrine is most pronounced in states of pathological vasodilation, such as sepsis [51]. Because CPB provokes vasoplegia, norepinephrine can restore physiological vascular resistance. On the other hand, vasoconstriction caused by norepinephrine may lead to ischemia in certain body areas (such as skeletal muscles, skin, and intestines) [52]. This could increase lactate production [53] and cause subsequent systemic acidosis, but the literature on this subject is equivocal [54]. Concerning kidney function, there are numerous studies that report the detrimental effect of norepinephrine on kidney function [55,56,57,58]. However, in some of these studies, there were high doses of norepinephrine administered, e.g., Azau et al. [56] administered doses as high as 0.37 µg/kg/min, and Vedel et al. [57] administered doses as high as 0.4 µg/kg/min. Bellomo et al. [59], in their review, argue that norepinephrine can be beneficial for kidney function if used properly. Norepinephrine should only be used to restore normal vascular resistance. It should not be used to artificially raise the systemic blood pressure if the pathology underlying hypotension is hypovolemia or inadequately low CO. In such instances, norepinephrine is likely to cause tissue hypoperfusion and exacerbate organ damage. Therefore, norepinephrine should be perceived as an adjuvant to intraoperative care and administered only after the patient is provided with an adequate CO and properly hydrated. Considering the results of previous studies [56,57], lower norepinephrine doses are encouraged (≤0.1 µg/kg/min).

#### 2.2.2. Epinephrine

Epinephrine is a catecholamine exerting a sympathomimetic effect on both alpha- and beta-adrenergic receptors [60]. Its pharmacodynamic properties comprise increase in heart rate, greater heart muscle contractility, increase in SVR (including renal vessels vasoconstriction), and bronchodilatation. In small doses, epinephrine has greater affinity to beta receptors, and in large doses it becomes selective to alpha receptors. As the heart muscle is excluded from circulation during the CPB, epinephrine has limited use during the procedure itself [61,62]. However, some authors found it beneficial for weaning from the CPB [63].

#### 2.2.3. Dopamine

Dopamine is a vasoactive agent which action is highly dependent on the dosage. In low doses (0.5–2 µg/kg/min), it dilates the visceral vessels, including the renal arteries. Moderate doses (2–10 µg/kg/min) increase cardiac output through augmented contractility and conductivity within the heart muscle. High doses (>10 µg/kg/min) increase SVR through action on the alpha- and beta-adrenergic receptors [64]. Theoretically, the vasodilatory effect on renal arteries improves the renal blood flow [65] and increases diuresis [66]. Nevertheless, the benefits of low-dose dopamine infusion during the CPB remain questionable [67]. As it would require high doses of dopamine to achieve its vasoconstrictive action, this catecholamine is not a first-choice drug in cardiac surgery.

#### 2.2.4. Fenoldopam

Fenoldopam is a dopamine receptor agonist that dilatates the peripheral arteries and lowers blood pressure [68]. Its action is most pronounced in renal arteries. Increased renal blood flow increases diuresis and natriuresis, thus augmenting the hypotensive action of this drug. Lee et al. [69] developed a simulation model proving that fenoldopam infusion is the most effective strategy for maintaining optimal renal perfusion during cardiac surgery. As vascular resistance usually decreases during the CPB, fenoldopam’s use in this setting is limited. However, if perfusion pressure remains high after the initiation of CPB, fenoldopam infusion can benefit both systemic circulation and renal blood flow.

#### 2.2.5. Nitroglycerine

Nitroglycerine is a vasodilatory drug that acts by donating a nitric group (NO), which relaxes the vascular smooth muscles [70]. Nitroglycerine dilatates small venous vessels (decreasing the preload) as well as resistant arteries (decreasing the afterload and systemic pressure). Venous vasculature dilatation has one more effect: increasing the volume of blood stored within this part of the circulatory system. The vascular resistance during the CPB usually decreases, partially due to endogenous nitric oxide release. For this reason, nitroglycerine is reserved for patients with increased vascular tone and perfusion pressure during the CPB. Nitroglycerine is also beneficial for kidney perfusion and increases diuresis [71].

#### 2.2.6. Nesiritide

Nesiritide is a recombinant human B-type natriuretic peptide (BNP), that causes vasodilation (including renal arteries), natriuresis, and RAA system inhibition [72]. Such mechanisms of action make it especially feasible for application in cardiac surgery. A meta-analysis conducted by Mitaka et al. [73] demonstrated that intraoperative BNP infusion increases the urine output and glomerular filtration rate, as well as reduces the postoperative serum creatinine levels. Also, a decreased time of ICU stay and hospital stay in patients treated with nesiritide was demonstrated in this study. Chen et al. [74] proved that patients with preoperative renal dysfunction also benefit from nesiritide infusion during cardiac surgery. Patients with preoperative left ventricular dysfunction had better kidney function and shorter hospital stay after intraoperative nesiritide infusion [75].

#### 2.2.7. Methylene Blue

Methylene blue is used during the CPB because of its potential to counteract the vasoplegic syndrome through the inhibition of nitric oxide synthase [76]. Kofler et al. [77] demonstrated an additive effect of methylene blue and other vasopressors in patients unresponsive to norepinephrine and vasopressin infusion. Similar conclusions were reached by other authors [78,79]. This proves that methylene blue is an effective agent against vasopressor-resistant vasoplegia during cardiac surgery. However, the impact of methylene blue on kidney function is debatable. In the study by Kofler et al., there was a significant rise in the postoperative serum creatinine concentration in patients receiving methylene blue compared to the control group. On the other hand, in the study by Mehaffey et al. [79], early administration of methylene blue reduced the incidence of postoperative renal failure. Considering the deleterious impact of vasoplegic syndrome and severe hypotension on kidney function, the use of methylene blue is a promising solution, but further investigation is needed to confirm its safety in terms of kidney function.

### 2.3. Fluid Balance and Osmotic Regulation

Water homeostasis is of vital importance to human health under any circumstances. Cardiac surgery, however, poses an exceptional challenge for maintaining fluid balance. Several factors can impact this balance: preoperative fasting, intraoperative evaporation from internal organs, high osmolality fluids infusion, and altered capillary fluid filtration.

Normal total water intake ranges from 2000–2700 mL/day for women and 2500–3700 mL/day for men, according to different health organizations worldwide [80]. When a patient reports to a hospital for scheduled admission, he or she usually comes in on an empty stomach for the purpose of laboratory tests. After admission, the patient is allowed to eat and drink according to his or her needs until the evening. Preoperative anxiety, however, causes some patients to reduce their food and fluid intake. The above factors considered, and after all night fasting, a significant percentage of the patients enter the operating theatre with various degrees of dehydration [81,82]. This type of dehydration can be classified as hypertonic dehydration, as it is caused by insufficient fluid intake with none to minimal solutes loss [83]. It results in intracellular fluid (ICF) movement to the extracellular compartment [84]. This allows for maintaining normal extracellular fluid (ECF) and plasma volume. Experimental studies indicate that in this kind of dehydration, the ICF deficits can persist even after three hours of ad libitum rehydration [85].

During cardiac surgery, two major factors exacerbate the patient’s hypertonic dehydration. First, the evaporation from mucosal membranes of the mediastinum and intrathoracic organs can account for 1000 mL of insensible water loss during routine CABG procedure [86]. The second factor is hypertonic fluids administration: the priming fluid of the CPB circuit and cardioplegic solution, which cause water to shift from the intracellular space to the extracellular space. The priming solution is highly hypertonic, reaching between 379 mOsm/l [87] and even 580 mOsm/l [88], according to data presented by different authors. The main reason for the high osmolality of this solution is the addition of mannitol. Intravenous mannitol is restricted to the extracellular compartment; it does not enter the cells [89]. After administration, a steady state is reached between the intravascular and extravascular mannitol concentration, shifting towards the vessels as mannitol is secreted with urine. It binds the water molecules that move freely between the intracellular and extracellular compartments, causing cells’ dehydration and osmotic diuresis. For these reasons, mannitol is known as a nephrotoxic agent. The effect of cardioplegia is similar, as standard cardioplegic solutions are also hypertonic (300–375 mOsm/l) [90]. Cardioplegia is administered to the ostia of the coronary arteries, but eventually, it reaches systemic circulation through the cardiac veins and venous cannula of the CPB circuit. The standard priming volume is 1400–1800 mL [91], and cardioplegia is 1100 mL for a single dose of Del Nido cardioplegia [92]. Gunnar et al. [93] demonstrated a significant increase in plasma osmolality after the commencement of CPB (322 ± 17 mOsm/kg). Hyperosmolality persists throughout the procedure (309 mOsm/kg in Gunnar’s population) and normalizes only on the first postoperative day. Hyperosmotic environment causes a water shift from the cells to the extracellular compartment, which may damage organs such as the kidneys or the brain and disrupt the immune system’s function [94]. Plasma osmolality of 320 mOsm/kg is reported to increase the risk of AKI. Dąbrowski et al. [94] also mention that hyperosmolality is especially detrimental to the kidneys as it forces osmotic diuresis within the glomerulus, thus reducing medullary blood flow.

One of the possible solutions to the problem of a high-volume priming fluid is using a miniaturized CPB circuit (mini-CPB). It is widely applied in the field of cardiac surgery [95,96], and apart from reducing the priming volume, it also reduces the need for blood transfusion, decreases the incidence of postoperative arrhythmias, and improves the overall treatment outcomes [97].

Another approach that can make the CPB more physiological and decrease the priming-related osmotic load is using albumins in the priming solution [98,99]. The need for a highly osmotic priming fluid is derived from the fact that this extra volume needs to be kept within the intravascular compartment. As discussed above, the priming fluid is also distributed within the interstitial space, but eventually, it returns to the vessels due to the osmotic gradient. Albumin exerts vessel-directed oncotic pressure [100] (as its concentration is higher in the serum than in the interstitial fluid) and could keep the extra volume within the vessels during the CPB. Russell et al. [99] demonstrated in their meta-analysis that using albumins in the priming solution improves the fluid balance and augments platelet preservation during the CPB. The same conclusion was reached by the expert consensus of Xiang et al. [101] One of the major issues with albumin usage is its high cost, and there are no randomized controlled trials demonstrating albumin’s superiority regarding postoperative outcomes such as mortality, ICU stay time, or organ dysfunction. All in all, albumin addition to the priming solution appears to be a promising perspective, but further scientific evidence is needed to confirm its suitability.

Another untrue notion amongst some medical professionals regarding hydration is that the patient does not need rehydration before cardiac surgery, as he or she will receive a lot of intravenous fluids during the procedure. As demonstrated above, the cardiac surgery patient is administered a significant amount of hyperosmolar fluids, which may cause cell damage and exacerbate hypertonic dehydration. Moreover, colloids (such as mannitol) may cause damage to the endothelial glycocalyx [102]. Therefore, to maintain normal fluid homeostasis, the patient must be intensively hydrated the day before the surgery to allow proper water distribution between the fluid compartments and sufficient cells hydration. It is safe to assume that on the day before the operation, the patients may reduce their fluid intake by half, so 1000 mL of fluids for women and 1500 mL for men appears to be a reasonable regimen based on their daily demand [80]. Needless to say, this concerns patients with no severe renal function impairment or congestive heart failure. In patients with normal kidney function and no relevant fluid congestion, the risk of fluid overload is very low, so the approach to fluid therapy in this instant can be liberal. There is no physiological advantage of intravenous rehydration over oral rehydration [103], but the patient’s compliance is obviously better with intravenous fluids administration. On the day of the operation, the sleep-related water deficit should be corrected before the patient is admitted to the operating theater. Intravenous-balanced crystalloids may be applied here, as well as special oral rehydration solutions [104,105]. Following the recommended 25–35 mL/kg/day maintenance fluid volume [106] and assuming an eight-hour sleep, an average 80 kg patient should be given around 800 mL of fluids. Another advantage of liberal fluid therapy in this setting is the increased clearance of metabolites eliminated through the kidneys (e.g., creatinine, urea). This is beneficial as it renders the filtration process easier for the kidneys during the challenging time of surgery and CPB.

The clinical significance of altered capillary fluid filtration is hard to estimate as it changes significantly with systemic and interstitial fluid pressure, according to Starling’s equation [107]. A decrease in the transcapillary filtration rate can be expected in patients with low MAP during the CPB. Increased interstitial fluid pressure (e.g., in congestive heart failure) may exacerbate this effect. On the other hand, an inflammatory response associated with CPB is believed by some authors to create a “capillary leakage”, which increases fluid extravasation during and after cardiac surgery [108]. However, the data on this subject is inconsistent, and some authors deny the existence of such a phenomenon [109]. Infusing hypertonic solutions increases the osmolality of the intravascular fluid, but it also decreases the intravascular oncotic pressure due to a greater dilution of albumin. In time, the ions and mannitol diffuse through the capillary barrier, changing the driving forces of filtration yet again. The net effect of these changes is hard to estimate. Disruption in the interstitial fluid production and absorption can lead to inadequate substance exchange between the vessels and the cells, as well as impaired metabolite clearance. Maintaining physiological MAP during the CPB and proper hydration seems reasonable to optimize the capillary filtration during cardiac surgery.

A fundamental issue concerning water balance during the CPB is intraoperative fluids administration. As mentioned above, priming fluid and cardioplegic solution are hyperosmotic and do not provide proper hydration. Moreover, they exacerbate hypertonic dehydration of the cells and pose a significant challenge for the kidneys. Therefore, a reasonable intraoperative fluid administration regimen is needed using balanced crystalloids. Needless to say, fluid infusion comes at the cost of decreasing the CaO_2_. Balance must always be kept between proper hydration and adequate iDO_2_. Where does the compromise lie, then? Considering the vast literature describing the detrimental effects of inadequate iDO214–18, it is not advised to prioritize hydration over maintaining an acceptable CaO_2_. A balanced crystalloid solution should be administered continuously during the procedure (large fluid boluses should be avoided), and provided a satisfying CaO_2_, the infusion rate should be guided by urine output. Hori et al. [110] defined oliguria during the CPB as diuresis < 1.5 mL/kg/h. Song et al. [111] found that diuresis < 4 mL/kg/h during the CPB is an independent AKI predictor. Considering a tremendous osmotic load associated with CPB commencement, the threshold of 4 mL/kg/h of urine output does not appear steep. Currently, closed-loop systems (e.g., Renal Guard^®^) adjust the intravenous fluids’ infusion rate to urine output in real time, which allows for a significant AKI incidence reduction [112].

Intraoperative hemofiltration is another procedure that significantly influences fluid balance during cardiac surgery. It allows for removing intravascular fluid, ions, and metabolites [8]. There are also strategies of using hemofiltration as a mean to remove proinflammatory cytokines during the CPB [113]. Unfortunately, there are no set indications for applying hemofiltration during the CPB. It is most commonly used in cases of severe hemodilution after the initiation of CPB or in severe hyperkaliemia. The therapeutic potential of hemofiltration is based on excessive intravascular fluid and metabolite removal. In normal settings, fluid overload and congestion cause increased venous pressure and interstitial oedema within the kidneys [114]. This impairs kidney function and makes removing excessive fluid through renal filtration impossible. However, when the CPB is initiated, there can be no intravascular fluid excess or congestion as the venous reservoir stores the excessive volume (assuming proper venous return). Therefore, fluid removal in this instance should be directed to achieve adequate hemoglobin concentration. Another factor is that the patient will eventually wean from the CPB, and most of the CPB circuit volume will be returned to circulation. In the case of fluid excess, hemofiltration should also be targeted to provide an adequate fluid balance in the post-CPB period. Intraoperative hypovolemia should be avoided though, as it has a proven detrimental impact on kidney function [115,116]. A further significant benefit of hemofiltration during the CPB is osmotic load and metabolite removal. This can facilitate kidney function after weaning from CPB. The literature reports no difference [117] or positive outcomes [118] of applying intraoperative hemofiltration regarding kidney function. This discrepancy may be due to the application of zero balance ultrafiltration in the study by Matata et al. [118] and the lack of data regarding fluid balance in the study by Kandil et al. [117]. All in all, the available data suggest that intraoperative hemofiltration can be beneficial for kidney function, especially in patients with decreased preoperative filtration rate. It should be applied to counteract excessive hemodilution and to remove osmotic load (e.g., when repeated cardioplegia infusions are needed). Hypovolemia must be avoided, and proper fluid balance must be maintained.

Furosemide is a loop diuretic agent, which can be utilized during cardiac surgery to promote diuresis and increase renal exertion of potassium, sodium, and chlorine ions [119]. The net effect of furosemide administration is similar to the use of hemofiltration: it decreases the intravascular volume and enhances ion removal. The vital difference is that furosemide does not improve creatinine and urea clearance, and furosemide-induced diuresis is not a sign of proper kidney function after cardiac surgery [120,121]. There are reports of furosemide’s positive impact on kidney function after cardiac surgery [112,122], which is probably related to maintaining a proper fluid balance. Other authors found no benefit of furosemide use in the perioperative period, including no difference in the need for renal replacement therapy after the surgery [123,124,125]. Zheng et al. [126] found that preoperative use of furosemide was associated with higher CSA-AKI risk.

### 2.4. Inflammatory Response

CPB is associated with a response of the immune system [11,127]. The main reasons for this are surgical trauma and blood exposure to large foreign surfaces (the CPB tubing system). Both cellular and humoral mechanisms are involved. Leukocytes roll along the CPB circuit’s artificial surface, eventually leading to their activation [128]. Similarly, contact between the artificial surface and C5a and C3d cytokines triggers the complement activation, which induces humoral inflammatory response [127]. Increased immune system activation decreases the total vascular resistance [22] and may change capillary permeability [108]. It is associated with worse clinical outcomes after cardiac surgery [129,130], including a higher incidence of AKI [131,132].

The ischemia-reperfusion injury is a particular form of inflammatory response that occurs after the blood flow is restored to previously ischemic tissues [133,134,135,136]. During ischemia, the ATP shortage results in a failure of the ATP-dependent ionic pumps and consequent intracellular sodium increase followed by an osmotic cells’ swelling. Calcium-related protease activation leads to the degradation of the cellular membrane. Gene expression is also disrupted in ischemic conditions. A plethora of genes are activated during hypoxia, leading to the increased translation of cytokines and proinflammatory mediators. During reperfusion, the influx of oxygen catalyzes the synthesis of reactive oxygen species (ROS). ROS are involved in a degradation of the lipid structures of the cellular membrane and subsequent eicosanoid synthesis, endothelial cells’ activation, and adhesion molecules expression. Neutrophils activated by the proinflammatory cytokines are a further source of ROS and proteases that augment tissue destruction.

Some strategies have been developed to decrease the immune response associated with cardiac surgery and CPB. These involve the use of heparin-coated CPB circuits [137], pharmacological interventions (e.g., polyethylene glycol, infliximab, sildenafil, nitro-linoleate, resveratrol, trimetazidine, and others) [135], and also aerobic exercise-induced preconditioning [138]. However, currently, there is no universal and widely accepted solution to this problem [130,139,140]. In light of this, it appears that the best strategy so far is to avoid organ ischemia during cardiac surgery to eliminate the IRI component of the generalized surgery-associated inflammatory response.

## 3. Conclusions

As demonstrated in this review, there are numerous alterations to kidney physiology during cardiac surgery. Considering the available scientific evidence, it is concluded that adequate kidney perfusion and fluid balance are the most critical factors determining postoperative kidney function. By adequate perfusion, one should understand perfusion with proper oxygen delivery and sufficient perfusion pressure. Maintaining the fluid balance is imperative for a normal kidney filtration process, which is essential for preserving the intra and postoperative kidney function. Vasopressors are an ally in maintaining normal kidney perfusion during cardiac surgery, provided they are used to correct the surgery-associated vasoplegia, not abnormalities of a different origin.

KEY POINTS:

- Provide an iDO_2_ no lower than 260–300 mL/min/m^2^ during the CPB
- Maintain MAP no lower than 70–90 mmHg during the whole procedure
- Use small doses of norepinephrine (preferably < 0.1 µg/kg/min) to correct the vasoplegia associated with general anesthesia and CPB
- Start hydrating the patient on the day preceding the surgery and continue hydration during the perioperative period
- During the CPB, keep the fluid balance that allows for a diuresis of ≥4 mL/kg/h (do not abuse furosemide!)

## 4. Future Directions

The review of the available literature regarding kidney function during cardiac surgery revealed that there is a need for a more holistic approach to this subject. As described above, there are several factors crucial for preserving kidney function during the CPB: adequate iDO_2_, high MAP, and a proper fluid balance. There is no evidence of the superiority of any of these factors over the others, hence the need to address them simultaneously in future studies in this area. Likewise, some other nephroprotective strategies could be implemented together to test their combined performance, e.g., mini-CPB with a heparin-coated tubing system and the addition of a nephroprotective pharmacological agent.

## Data Availability

There is no additional data for this study.

## References

[1] Veluz J.S., Leary M.C. (2017). Cerebrovascular Complications of Cardiac Surgery. Primer on Cerebrovascular Diseases.

[2] Schurle A., Koyner J.L. (2021). CSA-AKI: Incidence, Epidemiology, Clinical Outcomes, and Economic Impact. J. Clin. Med..

[3] Yan Y., Gong H., Hu J., Wu D., Zheng Z., Wang L., Lei C. (2023). Perioperative Parameters-Based Prediction Model for Acute Kidney Injury in Chinese Population Following Valvular Surgery. Front. Cardiovasc. Med..

[4] Harky A., Joshi M., Gupta S., Yi Teoh W., Gatta F., Snosi M. (2020). Acute Kidney Injury Associated with Cardiac Surgery: A Comprehensive Literature Review. Braz. J. Cardiovasc. Surg..

[5] Alghamdi A.A., Aqeeli M.O., Alshammari F.K., Altalhi S.M., Bajebair A.M., Al-Ebrahim K.E. (2022). Cardiac Surgery-Associated Acute Kidney Injury (CSA-AKI) in Adults and Pediatrics; Prevention Is the Optimal Management. Heart Surg. Forum..

[6] Sun C., Chen D., Jin X., Xu G., Tang C., Guo X., Tang Z., Bao Y., Wang F., Shen R. (2023). Association between Acute Kidney Injury and Prognoses of Cardiac Surgery Patients: Analysis of the MIMIC-III Database. Front. Surg..

[7] Cho J.S., Shim J.K., Lee S., Song J.W., Choi N., Lee S., Kwak Y.L. (2021). Chronic Progression of Cardiac Surgery Associated Acute Kidney Injury: Intermediary Role of Acute Kidney Disease. J. Thorac. Cardiovasc. Surg..

[8] Sarkar M., Prabhu V. (2017). Basics of Cardiopulmonary Bypass. Indian J. Anaesth..

[9] Melly L., Torregrossa G., Lee T., Jansens J.L., Puskas J.D. (2018). Fifty Years of Coronary Artery Bypass Grafting. J. Thorac. Dis..

[10] Shaefi S., Mittel A., Loberman D., Ramakrishna H. (2019). Off-Pump Versus On-Pump Coronary Artery Bypass Grafting—A Systematic Review and Analysis of Clinical Outcomes. J. Cardiothorac. Vasc. Anesth..

[11] Day J.R.S., Taylor K.M. (2005). The Systemic Inflammatory Response Syndrome and Cardiopulmonary Bypass. Int. J. Surg..

[12] De La Hoz M.A., Rangasamy V., Bastos A.B., Xu X., Novack V., Saugel B., Subramaniam B. (2022). Intraoperative Hypotension and Acute Kidney Injury, Stroke, and Mortality during and Outside Cardiopulmonary Bypass: A Retrospective Observational Cohort Study. Anesthesiology.

[13] Machin D., Allsager C. (2006). Principles of Cardiopulmonary Bypass. Contin. Educ. Anaesth. Crit. Care Pain..

[14] Lee Y., Kim S.H., Hwang H.Y., Sohn S.H., Choi J.W., Kim K.H. (2021). Perfusion Parameters during Cardiopulmonary Bypass as a Predictor of Acute Kidney Injury after Aortic Valve Replacement. Acute. Crit. Care.

[15] Mukaida H., Matsushita S., Kuwaki K., Inotani T., Minami Y., Saigusa A., Amano A. (2019). Time–Dose Response of Oxygen Delivery during Cardiopulmonary Bypass Predicts Acute Kidney Injury. J. Thorac. Cardiovasc. Surg..

[16] de Somer F., Mulholland J.W., Bryan M.R., Aloisio T., Van Nooten G.J., Ranucci M. (2011). O2 Delivery and CO2 Production during Cardiopulmonary Bypass as Determinants of Acute Kidney Injury: Time for a Goal-Directed Perfusion Management?. Crit Care.

[17] Ranucci M., Johnson I., Willcox T., Baker R.A., Boer C., Baumann A., Justison G.A., de Somer F., Exton P., Agarwal S. (2018). Goal-Directed Perfusion to Reduce Acute Kidney Injury: A Randomized Trial. J. Thorac. Cardiovasc. Surg..

[18] Srey R., Rance G., Shapeton A.D., Leissner K.B., Zenati M.A. (2019). A Quick Reference Tool for Goal-Directed Perfusion in Cardiac Surgery. J. Extra. Corpor. Technol..

[19] Daniels S.R., Kimball T.R., Khoury P., Witt S., Morrison J.A. (1996). Correlates of the Hemodynamic Determinants of Blood Pressure. Hypertension.

[20] Kristof A.S., Magder S. (1999). Low Systemic Vascular Resistance State in Patients Undergoing Cardiopulmonary Bypass. Crit. Care Med..

[21] Trammel J.E., Sapra A. (2022). Physiology, Systemic Vascular Resistance.

[22] Ltaief Z., Ben-Hamouda N., Rancati V., Gunga Z., Marcucci C., Kirsch M., Liaudet L. (2022). Vasoplegic Syndrome after Cardiopulmonary Bypass in Cardiovascular Surgery: Pathophysiology and Management in Critical Care. J. Clin. Med..

[23] Management of Cardiopulmonary Bypass—UpToDate. https://www.uptodate.com/contents/management-of-cardiopulmonary-bypass/print.

[24] Barral J.-P., Croibier A. (2011). Circulatory Physiology. Visc. Vasc. Manip..

[25] Kouz K., Hoppe P., Briesenick L., Saugel B. (2020). Intraoperative Hypotension: Pathophysiology, Clinical Relevance, and Therapeutic Approaches. Indian J. Anaesth..

[26] Tsikas D., Jordan J., Engeli S. (2015). Blood Pressure-Lowering Effects of Propofol or Sevoflurane Anaesthesia Are Not Due to Enhanced Nitric Oxide Formation or Bioavailability. Br. J. Clin. Pharmacol..

[27] Watso J.C., Huang M., Belval L.N., Cimino F.A., Jarrard C.P., Hendrix J.M., Hinojosa-Laborde C., Crandall C.G. (2022). Low-Dose Fentanyl Reduces Pain Perception, Muscle Sympathetic Nerve Activity Responses, and Blood Pressure Responses during the Cold Pressor Test. Am. J. Physiol. Regul. Integr. Comp. Physiol..

[28] Moore P.G., Quail A.W., Cottee D.B.F., McIlveen S.A., White S.W. (2000). Effect of Fentanyl On Baroreflex Control Of Circumflex Coronary Conductance. Clin. Exp. Pharmacol. Physiol..

[29] Şahin A.S., Duman A., Atalik E.K., Ögün C.Ö., Şahin T.K., Erol A., Özergin U. (2005). The Mechanisms of the Direct Vascular Effects of Fentanyl on Isolated Human Saphenous Veins in Vitro. J. Cardiothorac. Vasc. Anesth..

[30] Cipolla M.J. (2009). Control of Cerebral Blood Flow. The Cerebral Circulation.

[31] Burke M., Pabbidi M.R., Farley J., Rom R.J. (2014). Molecular Mechanisms of Renal Blood Flow Autoregulation. Curr. Vasc. Pharmacol..

[32] Kida Y. (2020). Peritubular Capillary Rarefaction: An Underappreciated Regulator of CKD Progression. Int. J. Mol. Sci..

[33] Navar L.G., Arendshorst W.J., Pallone T.L., Inscho E.W., Imig J.D., Bell P.D. (2008). The Renal Microcirculation. Compr. Physiol..

[34] Martini A.G., Danser A.H.J. (2017). Juxtaglomerular Cell Phenotypic Plasticity. High. Blood Press. Cardiovasc. Prev..

[35] Jefferson J.A., Thurman J.M., Schrier R.W. (2010). Pathophysiology and Etiology of Acute Kidney Injury. Comprehensive Clinical Nephrology.

[36] Brzozowski T. (2019). Konturek. Fizjol. Czlowieka.

[37] Fountain J.H., Kaur J., Lappin S.L. (2023). Physiology, Renin Angiotensin System.

[38] Dalal R., Bruss Z.S., Sehdev J.S. (2022). Physiology, Renal Blood Flow and Filtration.

[39] Denton K.M., Anderson W.P., Sinniah R. (2000). Effects of Angiotensin II on Regional Afferent and Efferent Arteriole Dimensions and the Glomerular Pole. Am. J. Physiol. Regul. Integr. Comp. Physiol..

[40] Lovshin J.A., Boulet G., Lytvyn Y., Lovblom L.E., Bjornstad P., Farooqi M.A., Lai V., Cham L., Tse J., Orszag A. (2018). Renin-Angiotensin-Aldosterone System Activation in Long-Standing Type 1 Diabetes. JCI Insight.

[41] Harrison-Bernard L.M. (2009). The Renal Renin-Angiotensin System. Am. J. Physiol. Adv. Physiol. Educ..

[42] Küllmar M., Saadat-Gilani K., Weiss R., Massoth C., Lagan A., Cortés M.N., Gerss J., Chawla L.S., Fliser D., Meersch M. (2021). Kinetic Changes of Plasma Renin Concentrations Predict Acute Kidney Injury in Cardiac Surgery Patients. Am. J. Respir. Crit. Care Med..

[43] Lannemyr L., Bragadottir G., Krumbholz V., Redfors B., Sellgren J., Ricksten S.E. (2017). Effects of Cardiopulmonary Bypass on Renal Perfusion, Filtration, and Oxygenation in Patients Undergoing Cardiac Surgery. Anesthesiology.

[44] Gewin L.S. (2021). Sugar or Fat? Renal Tubular Metabolism Reviewed in Health and Disease. Nutrients.

[45] Duke J. (2011). Renal Function and Anesthesia. Anesthesia Secrets. Anesthesia Secrets.

[46] Feher J. (2017). Functional Anatomy of the Kidneys and Overview of Kidney Function. Quantitative Human Physiology.

[47] Kennedy-Lydon T.M., Crawford C., Wildman S.S.P., Peppiatt-Wildman C.M. (2013). Renal Pericytes: Regulators of Medullary Blood Flow. Acta Physiol..

[48] Zoccali C., Mallamaci F., Finocchiaro P. (2002). Atherosclerotic Renal Artery Stenosis: Epidemiology, Cardiovascular Outcomes, and Clinical Prediction Rules. J. Am. Soc. Nephrol..

[49] Dobrek L. (2021). An Outline of Renal Artery Stenosis Pathophysiology—A Narrative Review. Life.

[50] Norepinephrine-StatPearls-NCBI Bookshelf. https://www.ncbi.nlm.nih.gov/books/NBK537259/.

[51] Foulon P., De Backer D. (2018). The Hemodynamic Effects of Norepinephrine: Far More than an Increase in Blood Pressure!. Ann. Transl. Med..

[52] Livesey M., Jauregui J.J., Hamaker M.C., Pensy R.A., Langhammer C.G., Eglseder W.A. (2020). Management of Vasopressor Induced Ischemia. J. Orthop..

[53] Qvisth V., Hagström-Toft E., Enoksson S., Bolinder J. (2008). Catecholamine Regulation of Local Lactate Production in Vivo in Skeletal Muscle and Adipose Tissue: Role of β-Adrenoreceptor Subtypes. J. Clin. Endocrinol. Metab..

[54] Khalil M.A., El Tahan M.R., Khidr A.M., Fallatah S., Abohamar A.D., Amer M.M., Makhdom F., El Ghoneimy Y., Al Bassam B., Alghamdi T. (2022). Effects of Norepinephrine Infusion during Cardiopulmonary Bypass on Perioperative Changes in Lactic Acid Level (Norcal). Perfusion.

[55] Huette P., Moussa M.D., Beyls C., Guinot P.G., Guilbart M., Besserve P., Bouhlal M., Mounjid S., Dupont H., Mahjoub Y. (2022). Association between Acute Kidney Injury and Norepinephrine Use Following Cardiac Surgery: A Retrospective Propensity Score-Weighted Analysis. Ann. Intensive Care.

[56] Azau A., Markowicz P., Corbeau J.J., Cottineau C., Moreau X., Baufreton C., Beydon L. (2014). Increasing Mean Arterial Pressure during Cardiac Surgery Does Not Reduce the Rate of Postoperative Acute Kidney Injury. Perfusion.

[57] Vedel A.G., Holmgaard F., Rasmussen L.S., Langkilde A., Paulson O.B., Lange T., Thomsen C., Olsen P.S., Ravn H.B., Nilsson J.C. (2018). High-Target versus Low-Target Blood Pressure Management during Cardiopulmonary Bypass to Prevent Cerebral Injury in Cardiac Surgery Patients: A Randomized Controlled Trial. Circulation.

[58] Al-Husinat L., Alsabbah A., Hmaid A.A., Athamneh R., Adwan M., Hourani M.N., Almakhadmeh S., Al Modanat Z.J., Ismail M.I.A., Varrassi G. (2023). Norepinephrine May Exacerbate Septic Acute Kidney Injury: A Narrative Review. J. Clin. Med..

[59] Bellomo R., Di Giantomasso D. (2001). Noradrenaline and the Kidney: Friends or Foes?. Crit. Care.

[60] Dalal R., Grujic D. (2007). Epinephrine. xPharm: The Comprehensive Pharmacology Reference.

[61] Linton N.W.F., Linton R.A.F. (2003). Haemodynamic Response to a Small Intravenous Bolus Injection of Epinephrine in Cardiac Surgical Patients. Eur. J. Anaesthesiol..

[62] Gillies M., Bellomo R., Doolan L., Buxton B. (2005). Bench-to-Bedside Review: Inotropic Drug Therapy after Adult Cardiac Surgery—A Systematic Literature Review. Crit. Care.

[63] Filho M.F.S., Barral M., Barrucand L., Cavalcanti I.L., Vercosa N. (2015). A Randomized Blinded Study of the Left Ventricular Myocardial Performance Index Comparing Epinephrine to Levosimendan Following Cardiopulmonary Bypass. PLoS ONE.

[64] Sonne J., Goyal A., Lopez-Ojeda W. (2022). Dopamine. Methods in Molecular Biology. https://www.ncbi.nlm.nih.gov/books/NBK535451/.

[65] Elkayam U., Ng T.M.H., Hatamizadeh P., Janmohamed M., Mehra A. (2008). Renal Vasodilatory Action of Dopamine in Patients With Heart Failure. Circulation.

[66] Pereira C.N., Machado F.R., Guimarães H.P., Resque Senna A.P., Gomes do Amaral J.L. (2004). Hemodynamics and Renal Function during Administration of Low-Dose Dopamine in Severely Ill Patients. Sao Paulo Med. J..

[67] Zeyneloglu P., Ozdemir H., Komurcu O., Bayraktar N., Sezgin A., Pirat A., Arslan G. (2012). Effects of Renal-Dose Dopamine on Renal Tubular Functions Following Coronary Artery Bypass Grafting Surgery. Crit. Care.

[68] Fenoldopam-StatPearls-NCBI Bookshelf. https://www.ncbi.nlm.nih.gov/books/NBK526058/.

[69] Lee C.J., Gardiner B.S., Smith D.W. (2020). A Cardiovascular Model for Renal Perfusion during Cardiopulmonary Bypass Surgery. Comput. Biol. Med..

[70] Kim K.H., Kerndt C.C., Adnan G., Schaller D.J. (2022). Nitroglycerin. Encyclopedia of Toxicology.

[71] Lee J.U. (2008). Nitric Oxide in the Kidney: Its Physiological Role and Pathophysiological Implications. Electrolytes Blood Press. E BP.

[72] Mathur A.G., Kairi J.K., Nayak B.B. (2005). Nesiritide—A New Agent for Acute Decompensated Heart Failure. Med. J. Armed. Forces India.

[73] Mitaka C., Kudo T., Haraguchi G., Tomita M. (2011). Cardiovascular and Renal Effects of Carperitide and Nesiritide in Cardiovascular Surgery Patients: A Systematic Review and Meta-Analysis. Crit. Care.

[74] Chen H.H., Sundt T.M., Cook D.J., Heublein D.M., Burnett J.C. (2007). Low Dose Nesiritide and the Preservation of Renal Function in Patients with Renal Dysfunction Undergoing Cardiopulmonary-Bypass Surgery A Double-Blind Placebo-Controlled Pilot Study. Circulation.

[75] Mentzer R.M., Oz M.C., Sladen R.N., Graeve A.H., Hebeler R.F., Luber J.M., Smedira N.G. (2007). Effects of Perioperative Nesiritide in Patients with Left Ventricular Dysfunction Undergoing Cardiac Surgery The NAPA Trial. J. Am. Coll. Cardiol..

[76] Naoum E.E., Dalia A.A., Roberts R.J., Devine L.T., Ortoleva J. (2022). Methylene Blue for Vasodilatory Shock in the Intensive Care Unit: A Retrospective, Observational Study. BMC Anesth..

[77] Kofler O., Simbeck M., Tomasi R., Hinske L.C., Klotz L.V., Uhle F., Born F., Pichlmaier M., Hagl C., Weigand M.A. (2022). Early Use of Methylene Blue in Vasoplegic Syndrome: A 10-Year Propensity Score-Matched Cohort Study. J. Clin. Med..

[78] Petermichl W., Gruber M., Schoeller I., Allouch K., Graf B.M., Zausig Y.A. (2021). The Additional Use of Methylene Blue Has a Decatecholaminisation Effect on Cardiac Vasoplegic Syndrome after Cardiac Surgery. J. Cardiothorac. Surg..

[79] Mehaffey J.H., Johnston L.E., Hawkins R.B., Charles E.J., Yarboro L., Kern J.A., Ailawadi G., Kron I.L., Ghanta R.K. (2017). Methylene Blue for Vasoplegic Syndrome after Cardiac Surgery: Early Administration Improves Survival. Ann. Thorac. Surg..

[80] Armstrong L.E., Johnson E.C. (2018). Water Intake, Water Balance, and the Elusive Daily Water Requirement. Nutrients.

[81] Rassam S.S., Counsell D.J. (2005). Perioperative Electrolyte and Fluid Balance. Contin. Educ. Anaesth. Crit. Care Pain.

[82] Löffel L.M., Engel D.A., Beilstein C.M., Hahn R.G., Furrer M.A., Wuethrich P.Y. (2021). Dehydration before Major Urological Surgery and the Perioperative Pattern of Plasma Creatinine: A Prospective Cohort Series. J. Clin. Med..

[83] Lacey J., Corbett J., Forni L., Hooper L., Hughes F., Minto G., Moss C., Price S., Whyte G., Woodcock T. (2019). A Multidisciplinary Consensus on Dehydration: Definitions, Diagnostic Methods and Clinical Implications. Ann. Med..

[84] Popkin B.M., D’Anci K.E., Rosenberg I.H. (2010). Water, Hydration and Health. Nutr. Rev..

[85] Nose H., Mack G.W., Shi X.R., Nadel E.R. (1988). Role of Osmolality and Plasma Volume During Rehydration in Humans. J. Appl. Physiol..

[86] Crerar-Gilbert A., Dewhurst A., Barnes S.C., Collinson P.O., Mcanulty G.R. (2001). Insensible Fluid Loss during Cardiac Surgery. Crit. Care.

[87] Darling E., Harris-Holloway S., Kern F.H., Ungerleider R., Jaggers J., Lawson S., Shearer I. (2000). Impact of Modifying Priming Components and Fluid Administration Using Miniaturized Circuitry in Neonatal Cardiopulmonary Bypass. Perfusion.

[88] Hrratsuka H. (1968). Clinical studies of hypothermic perfusion with hemodilution technique, especially its influence on water and electro-lytes changes, and renal function. Nagoya J. Med. Sci.

[89] Tenny S., Patel R., Thorell W. (2022). Mannitol.

[90] Bradić J., Andjić M., Novaković J., Jeremić N., Jakovljević V. (2023). Cardioplegia in Open Heart Surgery: Age Matters. J. Clin. Med..

[91] Hallward G., Hall R. (2009). Priming Solutions for Cardiopulmonary Bypass Circuits. Cardiopulmonary Bypass.

[92] Kim K., Ball C., Grady P., Mick S. (2014). Use of Del Nido Cardioplegia for Adult Cardiac Surgery at the Cleveland Clinic: Perfusion Implications. J. Extra. Corpor. Technol..

[93] Malmqvist G., Claesson Lingehall H., Appelblad M., Svenmarker S. (2018). Cardiopulmonary Bypass Prime Composition: Beyond Crystalloids versus Colloids. Perfusion.

[94] Dabrowski W., Siwicka-Gieroba D., Robba C., Bielacz M., Sołek-Pastuszka J., Kotfis K., Bohatyrewicz R., Jaroszyński A., Malbrain M.L.N.G., Badenes R. (2021). Potentially Detrimental Effects of Hyperosmolality in Patients Treated for Traumatic Brain Injury. J. Clin. Med..

[95] Alsatli R.A. (2012). Mini Cardiopulmonary Bypass: Anesthetic Considerations. Anesth. Essays. Res..

[96] Momin A., Sharabiani M., Mulholland J., Yarham G., Reeves B., Anderson J., Angelini G. (2013). Miniaturized Cardiopulmonary Bypass: The Hammersmith Technique. J. Cardiothorac. Surg..

[97] Cheng T., Barve R., Cheng Y.W.M., Ravendren A., Ahmed A., Toh S., Goulden C.J., Harky A. (2021). Conventional versus Miniaturized Cardiopulmonary Bypass: A Systematic Review and Meta-Analysis. JTCVS Open.

[98] Rauf A., Joshi R.K., Aggarwal N., Agarwal M., Kumar M., Dinand V., Joshi R. (2021). Effect of Albumin Addition to Cardiopulmonary Bypass Prime on Outcomes in Children Undergoing Open-Heart Surgery (EACPO Study)—A Randomized Controlled Trial. World J. Pediatr. Congenit. Heart Surg..

[99] Russell J.A., Navickis R.J., Wilkes M.M. (2004). Albumin Versus Crystalloid for Pump Priming in Cardiac Surgery: Meta-Analysis of Controlled Trials. J. Cardiothorac. Vasc. Anesth..

[100] Munoz A.C., Jain N.K., Gupta M. (2023). Albumin Colloid.

[101] Xiang F., Huang F., Huang J., Li X., Dong N., Xiao Y., Zhao Q., Xiao L., Zhang H., Zhang C. (2023). Consensus Statement Quick Response Code: Expert Consensus on the Use of Human Serum Albumin in Adult Cardiac Surgery. Chin. Med. J..

[102] Milford E.M., Reade M.C. (2019). Resuscitation Fluid Choices to Preserve the Endothelial Glycocalyx. Crit. Care.

[103] Atherly-John Y.C., Cunningham S.J., Crain E.F. (2002). A Randomized Trial of Oral vs Intravenous Rehydration in a Pediatric Emergency Department. Arch. Pediatr. Adolesc. Med..

[104] Taniguchi H., Sasaki T., Fujita H. (2011). Oral Rehydration Therapy for Preoperative Fluid and Electrolyte Management. Int. J. Med. Sci..

[105] Dalal K.S., Rajwade D., Suchak R. (2010). “Nil per Oral after Midnight”: Is It Necessary for Clear Fluids?. Indian J. Anaesth..

[106] (2017). CG174.

[107] Scallan J., Huxley V.H., Korthuis R.J. (2010). Fluid Movement Across the Endothelial Barrier.

[108] Hamada Y., Kawachi K., Tsunooka N., Nakamura Y., Takano S., Imagawa H. (2004). Capillary Leakage in Cardiac Surgery with Cardiopulmonary Bypass. Asian Cardiovasc. Thorac. Ann..

[109] Tassani P., Schad H., Winkler C., Bernhard A., Ettner U., Braun S.L., Eising G.P., Kochs E., Lange R., Richter J.A. (2002). Capillary Leak Syndrome after Cardiopulmonary Bypass in Elective, Uncomplicated Coronary Artery Bypass Grafting Operations: Does It Exist?. J. Thorac. Cardiovasc. Surg..

[110] Hori D., Katz N.M., Fine D.M., Ono M., Barodka V.M., Lester L.C., Yenokyan G., Hogue C.W. (2016). Defining Oliguria during Cardiopulmonary Bypass and Its Relationship with Cardiac Surgery–Associated Acute Kidney Injury. BJA Br. J. Anaesth..

[111] Song Y., Kim D.W., Kwak Y.L., Kim B.S., Joo H.M., Ju J.W., Yoo Y.C. (2016). Urine Output During Cardiopulmonary Bypass Predicts Acute Kidney Injury After Cardiac Surgery: A Single-Center Retrospective Analysis. Medicine.

[112] Luckraz H., Giri R., Wrigley B., Nagarajan K., Senanayake E., Sharman E., Beare L., Nevill A. (2021). Balanced Forced-diuresis as a Renal Protective Approach in Cardiac Surgery: Secondary Outcomes of Electrolyte Changes. J. Card Surg..

[113] Atan R., Crosbie D.C.A., Bellomo R. (2013). Renal Failure Techniques of Extracorporeal Cytokine Removal: A Systematic Review of Human Studies Techniques of Extracorporeal Cytokine Removal: A Systematic Review of Human Studies. Ren. Fail.

[114] Selewski D.T., Goldstein S.L. (2018). The Role of Fluid Overload in the Prediction of Outcome in Acute Kidney Injury. Pediatr. Nephrol..

[115] Yu Y., Li C., Zhu S., Jin L., Hu Y., Ling X., Miao C., Guo K. (2023). Diagnosis, Pathophysiology and Preventive Strategies for Cardiac Surgery-Associated Acute Kidney Injury: A Narrative Review. Eur. J. Med. Res..

[116] Udzik J., Waszczyk A., Safranow K., Biskupski A., Majer K., Kwiatkowski S., Kwiatkowska E. (2021). Assessment and Prognosis in CSA-AKI Using Novel Kidney Injury Biomarkers: A Prospective Observational Study. Biology.

[117] Kandil O.A., Motawea K.R., Darling E., Riley J.B., Shah J., Elashhat M.A.M., Searles B., Aiash H. (2021). Ultrafiltration and Cardiopulmonary Bypass Associated Acute Kidney Injury: A Systematic Review and Meta-Analysis. Clin. Cardiol..

[118] BM M. (2017). The Risk-Adjusted Impact of Intraoperative Hemofiltration on Real-World Outcomes of Patients Undergoing Cardiac Surgery. J. Clin. Nephrol..

[119] Khan T.M., Patel R., Siddiqui A.H. (2023). Furosemide.

[120] Joannidis M., Klein S.J., Ostermann M. (2019). 10 Myths about Frusemide. Intensiv. Care Med..

[121] Ho K.M., Power B.M. (2010). Benefits and Risks of Furosemide in Acute Kidney Injury. Anaesthesia.

[122] Fakhari S., Bavil F.M., Bilehjani E., Abolhasani S., Mirinazhad M., Naghipour B. (2017). Prophylactic Furosemide Infusion Decreasing Early Major Postoperative Renal Dysfunction in On-Pump Adult Cardiac Surgery: A Randomized Clinical Trial. Res. Rep. Urol..

[123] Heringlake M., Klaus S., Bahlmann L., Gosch U., Schumacher J., Schmucker P. (2002). The Effects of a Single Dose of Furosemide on Urine Flow, Fluid Balance, and the Course of Plasma Creatinine during Cardiac Surgery. Crit. Care.

[124] Mahesh B., Yim B., Robson D., Pillai R., Ratnatunga C., Pigott D. (2008). Does Furosemide Prevent Renal Dysfunction in High-Risk Cardiac Surgical Patients? Results of a Double-Blinded Prospective Randomised Trial. Eur. J. Cardio-Thorac. Surg..

[125] Gandhi A., Husain M., Salhiyyah K., Raja S.G. (2012). Does Perioperative Furosemide Usage Reduce the Need for Renal Replacement Therapy in Cardiac Surgery Patients?. Interact Cardiovasc. Thorac. Surg..

[126] Zheng H., Liu L., Fan G., Liu Z., Wang Z., Chang B. (2021). Preoperative Use of Furosemide May Increase the Incidence of Acute Kidney Injury after Coronary Artery Bypass Grafting: A Propensity Score-Matched Study. Gen. Thorac. Cardiovasc. Surg..

[127] Diegeler A., Doll N., Rauch T., Haberer D., Walther T., Falk V., Gummert J., Autschbach R., Mohr F.-W. (2000). Humoral Immune Response During Coronary Artery Bypass Grafting. Circulation.

[128] Rossaint J., Berger C., van Aken H., Scheld H.H., Zahn P.K., Rukosujew A., Zarbock A. (2012). Cardiopulmonary Bypass during Cardiac Surgery Modulates Systemic Inflammation by Affecting Different Steps of the Leukocyte Recruitment Cascade. PLoS ONE.

[129] Warltier D.C., Laffey J.G., Boylan J.F., Cheng D.C. (2002). The Systemic Inflammatory Response to Cardiac SurgeryImplications for the Anesthesiologist. Anesthesiology.

[130] Squiccimarro E., Stasi A., Lorusso R., Paparella D. (2022). Narrative Review of the Systemic Inflammatory Reaction to Cardiac Surgery and Cardiopulmonary Bypass. Artif. Organs..

[131] Milne B., Gilbey T., De Somer F., Kunst G. (2023). Adverse Renal Effects Associated with Cardiopulmonary Bypass. Perfusion.

[132] Presta P., Bolignano D., Coppolino G., Serraino F., Mastroroberto P., Andreucci M., Fuiano G. (2021). Antecedent ACE-Inhibition, Inflammatory Response, and Cardiac Surgery Associated Acute Kidney Injury. Rev. Cardiovasc. Med..

[133] Cowled P., Fitridge R. (2011). Pathophysiology of Reperfusion Injury. Mechanisms of Vascular Disease: A Reference Book for Vascular Specialists.

[134] Linfert D., Chowdhry T., Rabb H. (2009). Lymphocytes and Ischemia-Reperfusion Injury. Transpl. Rev..

[135] Soares R.O.S., Losada D.M., Jordani M.C., Évora P., Castro-E-Silva O. (2019). Ischemia/Reperfusion Injury Revisited: An Overview of the Latest Pharmacological Strategies. Int. J. Mol. Sci..

[136] Malek M., Nematbakhsh M. (2015). Renal Ischemia/Reperfusion Injury; from Pathophysiology to Treatment. J. Ren. Inj. Prev..

[137] Hein E., Munthe-Fog L., Thiara A.S., Fiane A.E., Mollnes T.E., Garred P. (2015). Heparin-Coated Cardiopulmonary Bypass Circuits Selectively Deplete the Pattern Recognition Molecule Ficolin-2 of the Lectin Complement Pathway in Vivo. Clin. Exp. Immunol..

[138] Borges J.P., da Silva Verdoorn K. (2017). Cardiac Ischemia/Reperfusion Injury: The Beneficial Effects of Exercise. Adv. Exp. Med. Biol..

[139] Bronicki R.A., Hall M. (2016). Cardiopulmonary Bypass-Induced Inflammatory Response: Pathophysiology and Treatment. Pediatr. Crit. Care Med..

[140] Kant S., Banerjee D., Sabe S.A., Sellke F., Feng J. (2023). Microvascular Dysfunction Following Cardiopulmonary Bypass Plays a Central Role in Postoperative Organ Dysfunction. Front. Med..

